# The Role of Emotional Responses in the VR Exhibition Continued Usage Intention: A Moderated Mediation Model

**DOI:** 10.3390/ijerph20065001

**Published:** 2023-03-12

**Authors:** Minglu Wang, Jong-Yoon Lee, Shanshan Liu, Lingling Hu

**Affiliations:** 1 School of Art, Qilu University of Technology, Jinan 250353, China; 2 School of Art, Sangmyung University, Cheonan 330-720, Republic of Korea; 3 School of Communication, Nanyang Institute of Technology, Nanyang 473004, China

**Keywords:** emotional responses, experience, psychological health, presence, continued usage intention

## Abstract

During COVID-19, many renowned galleries and art fairs used Virtual Reality (VR) exhibitions for art information dissemination and online displays. To avoid the risks of offline viewing of exhibitions, users can access a web-based VR exhibition platform for remote appreciation of artworks, gaining a rich art experience and thus contributing to physical and mental health. The reasons affecting users’ continued usage intentions are not clear enough in the existing studies of VR exhibitions. Therefore, further studies are needed. This paper explores the relationship between users’ escapist experience, aesthetic experience, presence, emotional responses, and continued usage intention through a survey of VR exhibition users. The survey data were collected from 543 users who had experienced the VR exhibition through an online survey website. The study results show that users’ continued usage intentions are influenced by escapist experience and aesthetic experience. Presence plays a mediating role in the influence of escapist experiences and aesthetic experiences on continued usage intention. Emotional responses play a moderating role in the impact of user experience on continued usage intention. This paper provides a theoretical reference for the study of the impact mechanism of continued usage intention of VR exhibitions from the perspective of mental health. In addition, this study enables VR exhibition platforms to better understand the emotional state of users during art experiences to create and share healthy aesthetic information that can contribute to the management and enhancement of mental health. At the same time, it provides valuable and innovative guidance solutions for the future development of VR exhibitions.

## 1. Introduction

In recent years, VR exhibitions have flourished and become popular as a new type of exhibition [1]. A VR exhibition is an exhibition held in a computerized virtual space based on online network technology and image digital technology [2]. According to the type of media, VR exhibitions are divided into web exhibitions, online exhibitions, digital exhibitions, and other forms [2,3]. Digital technology has become a focal point for health interventions and healthcare, and plays an important role in global public health, with the function of delivering health information and increasing the mental health of users [4]. However, the global spread of COVID-19 has led to a health crisis, causing negative emotions and affecting the physical and mental health of people. The application of interactive and entertainment functions of digital technology also helps individuals to relax and get rid of the psychological worries and pains in their lives, and to restore the coordinated functioning of their psychological functions. In particular, with the unique virtual experience properties of digital technology, VR exhibitions allow for remote viewing and interactive artwork. More and more users experience art through VR exhibitions, satisfying their cultural and artistic needs, and generating positive emotions and a sense of wellbeing. In other words, the appreciation and experience of art have a positive impact on human psychological functions [5,6]. Research has shown that the rapid growth and expansion of the VR exhibition market has made up for the lack of offline exhibitions and created a whole new world of exhibition culture [7,8]. Therefore, it is also important to study the factors influencing the continued usage intention of VR exhibitions.

Users can enjoy art exhibition works from around the world anytime and anywhere through VR exhibitions, to interact and share in the virtual environment, generating escapist experiences and aesthetic experiences [9]. Research has shown that user experience is closely related to continued usage intention [10,11]. When users get a better experience, they will have continued usage intention [12,13]. However, the research literature on VR exhibitions during COVID-19 is uncommon. The mechanisms by which users’ escapist and aesthetic experiences influence continued usage intentions are unclear. In addition, because users experience virtual environments with different psychological states and generate different intentions and behaviors with potential moderating factors, it is necessary to conduct empirical studies focusing on the actual impact of emotional reactions in the process of art experience.

The main objective of our study was: first, to determine the effects of escapist and aesthetic experiences on continued usage intention. Second, to explore the mediating role of presence in escapist and aesthetic experiences on continued usage intention and the moderating role of emotional responses on continued usage intention. This study provides new information on the psychological and health cognitive dimensions of user experience during the COVID-19 pandemic, and informs the use and development of digital technologies in global public health.

## 2. Theoretical Framework and Hypothesis

### 2.1. Experience Economy Theory

Pine and Gilmore developed a theory of the experience economy and defined experience as an activity in which the individual is fully engaged at the level of the body and mind [14]. They argue that experience can be analyzed in two dimensions: consumer participation in experience and connectedness with experience. As a science of human psychology and behavior, psychology also pays attention to user experience and economic activities [15]. Prior research has analyzed psychological factors in the process of experience economy from a psychological perspective, in particular, the decision-making process of user behavior in specific situations and the psychological factors that influence these decisions [16,17]. In addition, the experience economy theory also applies to the VR exhibition field [18]. Users can enjoy artworks through VR exhibitions, obtain art information, feel the visual impact of artworks, achieve a pleasant aesthetic experience, and generate a positive psychological state [2]. According to prior research, experiential refers to the extent to which individuals participate in VR exhibitions, and it includes diverse domains such as escapist and aesthetic experiences. A good experience improves the continued usage intention of users [9,19,20]. The escapist experience means that the user is immersed in the VR exhibition and actually enjoys the artwork, resulting in a mental state of concentration and enjoyment. The aesthetic experience is a fundamental component of VR exhibitions because VR exhibitions need to first engage users through aesthetic elements in order to provide an immersive art appreciation experience that leads to the perception of escapism [8]. Based on prior research, this study investigates the emotional responses and psychological factors of VR exhibition users using the framework of experience economy theory based on the characteristics of VR exhibitions and the purpose of the study.

### 2.2. The Impact of Escapist Experience on Continued Usage Intention

As a result of COVID-19, personal outings and activities have become increasingly difficult, and recreational and social activities have been reduced. People generally want to experience and interact through online media. They are meeting art and cultural needs through the use of VR exhibitions [21,22]. Escapist experience is a state of detachment from reality, the degree to which the individual is completely immersed in the object of experience [23,24]. Escapist experience is often described as a feeling of immersion in a new environment and separation from the everyday environment [25,26]. Users are taken out of the real space through the escapist experience and enter a virtual space where they can freely move [27]. As the user escapist experience increases, it is possible to effectively interact in the new environment [19,28]. During COVID-19, the escapist experience as a participation act of VR-integrated cultural space allows users to experience a peculiar process of merging with the virtual environment, gaining an enjoyable and memorable artistic feeling [29]. In this case, the user will gain an escapist experience through continued usage. When users feel detached from reality, they increase continued usage intention and use continued usage as an effective way to gain escapist experience [30,31]. Therefore, this study hypothesized that:

**H1:**  *Escapist experience would have an impact on continued usage intention.*

### 2.3. The Impact of Aesthetic Experience on Continued Usage Intention

Aesthetic experience is the experience of discovering beauty through objects, being moved by beauty, and gaining it through visual or cognitive levels of perception [32,33]. Most psychological perspectives on aesthetic experience consider it to be the result of the coordination of different mental processes, such as perception, imagination, thought, and emotion [5,34]. Aesthetic experience can be considered the degree of perceived artistry that the experiencing object possesses through indirect experience [35,36]. The aesthetic perceptions of users differ depending on the experience environment, which provides various visual, auditory, and aesthetic elements that trigger various perceptions [8]. VR exhibitions present works of art in themselves with high ornamental value. The artistic design and exhibition content in the exhibition space, as the core visual appreciation part, will pay more attention to the user’s aesthetic experience [10,37]. In an aesthetic experience, users feel positive emotions and pleasure when they like to immerse themselves in a virtual space and obtain aesthetic information conveyed by the environment and artwork that can facilitate the sensory stimulation required for an aesthetic experience [38,39]. Therefore, to obtain a pleasant experience and ideal culture and art, users will increase their continued usage intention. From this, we can see that aesthetic experience is a key factor in determining continued usage intention. This study hypothesized that:

**H2:**  *Aesthetic experience would have an impact on continued usage intention.*

### 2.4. The Mediating Impact of Presence

Presence is a user’s perceptual state or psychological reaction in an immersive virtual experience in which the user is very immersed [40,41]. Presence can be thought of as the subjective feeling that the user has forgotten the physical situation or location, but exists in a virtual environment created by a medium such as a computer or television [42,43]. Presence is a necessary variable for user behavior in a virtual environment [44,45]. Users increase the use of virtual media to feel the escapist experience and aesthetic experience [31]. In this process, individuals gain escapist experience by experiencing virtual spaces and environments [29]. In particular, the emergence of VR exhibitions has addressed the need for users to experience culture and art in the virtual world [46]. VR exhibitions allow users to immerse themselves in the art environment and interact with the exhibition program and other users [47]. Users can have an escapist and aesthetic experience when using VR exhibitions because they can immerse themselves in the artistic environment and forget about the real world around them [9]. By participating and interacting with the VR exhibition art space, enjoying artworks of different expressions and styles, and perceiving rich aesthetic elements, individuals gain aesthetic experiences and generate positive perceptual experiences [8]. In the process of using a VR exhibition, the individual has a multi-layered virtual experiential, which allows the user to immerse in the exhibition environment and generate presence. Thus, escapist and aesthetic experience will enhance presence. Presence allows users to appreciate art more authentically, enabling them to enhance their enjoyment and pleasure, resulting in higher continued usage intention [48,49]. During COVID-19, there were relatively few studies on the factors influencing users’ intention to use the virtual experience, and the VR exhibition experience can generate higher presences compared to the traditional exhibition medium. In particular, features such as ease of use, high definition, high stereo, and high sound quality create a higher level of immersion and positive emotion in the user’s enjoyment. In other words, the higher the presence generated during the experience, the stronger the intention to use it. Based on the above study, we proposed the following hypothesis:

**H3:**  *The escapist experience has an impact on the continued usage intention of the VR exhibition through presence.*

**H4:**  *The aesthetic experience has an impact on the continued usage intention of the VR exhibition through presence.*

### 2.5. Moderating Role of Emotional Responses

In VR exhibitions, emotional responses are described as important internal responses to the user’s interaction with the virtual environment. Specifically, users’ emotional responses are described as psychological phenomena or are used as a basis for explaining behavioral decisions [50]. These emotional responses are directly related to user satisfaction and thus influence the user’s decision to use the product [51,52]. In previous studies, emotional responses have been determined as an important factor influencing users’ continued usage intentions. The emotional response represents the user’s emotions, needs, and sensory reactions, and is of great importance in the actual experience and use of the product [53,54]. Research shows that when users generate good emotional responses, the product will be more positively accepted by users, and when users do not generate more positive emotional responses, it will reduce users’ favorable perception of the product and lower their continued usage intention [55,56]. Although users’ escapist and aesthetic experiences have an impact on continued usage intention, it may reduce users’ experience and usage when their virtual experience generates low positive emotional responses. Continued usage intention of users is lower for products with lower emotional responses. A good emotional response enhances the continued usage intention of users [42,57]. During COVID-19, there were fewer studies on user emotional responses to VR exhibitions. Compared with traditional exhibition media, VR exhibitions are more likely to produce strong emotional responses such as pleasure, excitement, and arousal, and users show more positive attitudes toward the continued usage of VR exhibitions [58]. Whether emotional responses enhance the effect of escapist and aesthetic experiences on continued usage intention is a question worthy of in-depth study. Therefore, this study hypothesized that:

**H5:**  *Emotional responses enhance the escapist experience’s impact on the VR exhibition’s continued usage intention.*

**H6:**  *Emotional responses enhance the aesthetic experience’s impact on the VR exhibition’s continued usage intention.*

**Figure 1:**
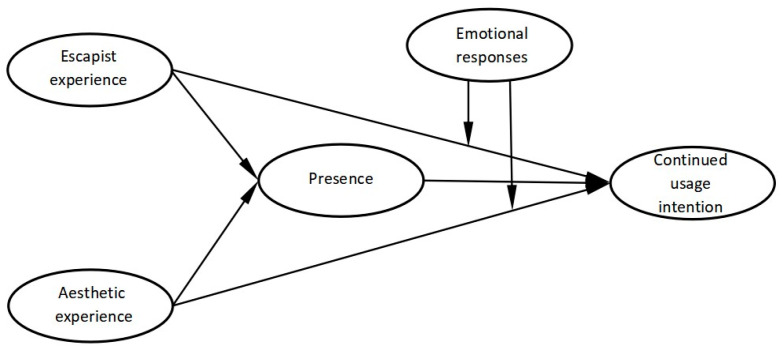
Research model.

In summary, the research model is developed in this paper (Figure 1).

## 3. Materials and Methods

### 3.1. Participants

This study conducted data collection using a questionnaire to test the research hypothesis and model. Specifically, we selected the relevant variables and questionnaire questions that fit the topic of this study based on the relevant prior studies on VR exhibitions or digital media adoption, and adopted the more mature ones that have been used in related fields. Before the survey, five experts reviewed the questionnaire to determine the appropriateness of the questionnaire items and exhibition content selection. Inappropriate items were modified based on the recommendations of the experts. The precautions taken before the survey, the way to experience, and the URL of the VR exhibition experience were explained in the introduction section of the questionnaire. We used Chinese as the main language for the questionnaire introduction and pre-survey. We chose users who had experienced VR exhibitions to conduct the survey. Users could directly click on the URL to enter the VR exhibition experience and participate in the questionnaire after the experience. This could bring users a more intuitive feeling and make it easier to understand. The average survey time for users to fill out the questionnaire was 23 min; if users had no experience or time to fill out the questionnaire, it was be deleted and was not considered a valid questionnaire. Finally, we selected the eligible questionnaires for further study. Specifically, the VR collection exhibition of the world-renowned Musée du Louvre, which has the highest rate of the display and is representative of the museum, was selected for investigation in this study. The works in the exhibition presented the transition and metamorphosis of European art from the Classical Age to the Renaissance, which is of great significance in the history of world art. In addition, a pre-survey of 85 VR exhibition users was conducted. The surveyed users indicated that the questionnaire was easy to complete. Based on the preliminary findings, no changes were needed to the questionnaire and the items were valid and reliable. The specific measurement items are presented in Table A1 in Appendix A. To collect the actual data for this study, 543 users of VR exhibitions in China participated in this study and filled out an informed consent form before completing the questionnaire. Information from the questionnaire was collected through the WENJUANXING data collection website. Specifically, these data were collected between 15 September and 29 October 2022.

### 3.2. Variable Measurement

#### 3.2.1. Escapist Experience

The escapist experience measure was modified from Kim and Park et al. escapist experience scale [59,60,61]. This scale includes 15 items (e.g., “Do you have a feeling of being in another place while experiencing the VR exhibition?”). The questionnaire was measured using five-point Likert items, with options ranging from strongly disagree (1) to strongly agree (5). The higher the score, the higher the degree of user escapist experience.

#### 3.2.2. Aesthetic Experience

This study used a modified aesthetic experience scale by Kong and Chang et al. [60,62]. This scale contains nine items (e.g., “I am very satisfied with the design style of the VR exhibition experience showroom”). The questionnaire options ranged from strongly disagree (1) to strongly agree (5). The higher the score, the higher the degree of the user’s aesthetic experience.

#### 3.2.3. Presence

According to Wang’s revision, the presence scale was used in this study (e.g., “ I felt that the exhibits in these virtual spaces were like real exhibits in front of my eyes.”) [2]. The scale has also been used in previous studies of VR exhibitions and is relatively accurate and mature, so it was also adopted for this study.

#### 3.2.4. Emotional Responses

The study was adapted from a scale developed by Lee and Cho (e.g., “ I felt pleasure and satisfaction when I had an art experience in the environment of a VR exhibition”) and has a high degree of accuracy and applicability [63]. The options in the questionnaire ranged from strongly disagree (1) to strongly agree (5).

#### 3.2.5. Continued Usage Intention

The questionnaire is based on the one developed by Shetu et al. (e.g., “ I am currently using the VR exhibit and will continue to use it”) [64]. The options in the questionnaire ranged from strongly disagree (1) to strongly agree (5).

### 3.3. Procedure

Studies involving human participants were reviewed and approved by the Academic Research Ethics Committee of Sangmyung University. In order to comply with academic ethics, approval and consent were obtained from the respondents prior to the survey. Participants provided written informed consent to participate in the study. Written informed consent needed to be obtained from the individual for any potentially identifiable data or images included in this study. When referring to individuals or companies, pseudonyms were used to protect the anonymity of the interviewees.

### 3.4. Data Analysis

SPSS 25.0 software was used for statistical analysis. First, descriptive statistics were performed for the main variables, and second, correlation analyses were performed. The effect of escapist experience and aesthetic experience on continued usage intention was analyzed by SPSS regression. In addition, the SPSS PROCESS macro model 5 was used to analyze the effect of presence on escapist experience and aesthetic experience, and continued usage intention was mediated and moderated [65]. SPSS PROCESS macro can perform mediating and moderating effects analysis by providing several specific models. The main objective was to assess the strength and importance of the linkages of the conceptual framework in order to analyze the interactions between the variables in the study and to reveal the direct and indirect effects between them.

## 4. Results

### Descriptive Statistics

Of the 543 valid survey samples, there were 285 females and 258 males. In addition, we examined the age of the users, divided into five interval groups (SD = 1.128). Skewness and kurtosis tests were performed in order to measure whether the obtained data had a normal distribution. Sample escapist experience mean = 3.23, skewness = −0.05, kurtosis = −0.40. Sample aesthetic experience mean = 3.25, skewness = −0.21, kurtosis = −0.64. Since the skewness and kurtosis values are between +1.96 and −1.96, the sample is normally distributed [2]. The report data shows that people aged 18−33 account for more than 70.90%, and 62.98% of the sample has a bachelor’s degree or above. Most people had experienced a VR exhibition on average once every 7 days. Table 1 shows the descriptive statistics of this study. The results of descriptive statistics and correlations between the main variables are summarized in Table 2.

Reliability tests were conducted in this study. Cronbach’s alpha is considered good if it is greater than 0.8 [3]. The results are shown in Table 2, and the variables are considered reliable. In addition, a correlation analysis was performed. Correlation analysis focuses on detecting the extent to which variables are closely related to each other, and this study uses Pearson correlation coefficients to measure this. The correlation is relatively high when Pearson is between 0.4 and 0.7 [2]. The results showed that the correlations of obtaining escapist experience, aesthetic experience, presence, and emotional response to continued usage intention were 0.489 **, 0.633 **, 0.480 **, and 0.481 **, respectively, and all four groups of variables were significantly correlated at the *p* < 0.01 level, while the correlation coefficients did not exceed 0.8. Therefore, there is no co-linearity between the four groups of variables and they are significantly correlated with continued usage intention and can continue to be analyzed.

Table 3 depicts the relationship between users’ escapist experience, aesthetic experience, and continued usage intention. Specifically, escapist experience has a significant impact on continued usage intention (β = 0.481, SE = 0.051, t = 12.767). In addition, aesthetic experience has a significant impact on continued usage intention (β = 0.374, SE = 0.045, t = 9.390). Both hypothesis 1 and hypothesis 2 were supported. Mediation analysis was used to examine the mediating role of presence between escapist experience, aesthetic experience, and continued usage intention. Tests were implemented using SPSS PROCESS macro model 5. Intermediate effects explain the strength of the direct and indirect effects of the conceptual model. In this study, a bootstrap sample of 5000 was designated to confirm indirect effects and they were analyzed with a 95% confidence interval. The estimated value of indirect effects was judged to be significant when the 95% confidence interval did not contain 0 [65]. Among them, all data were standardized. There was a significant mediating effect of presence (Table 3). We suggest a mediating role for the presence between escapist experience and continued usage intention (β = 0.335, bootstrap 95% confidence interval = 5000 for [0.235, 0.442], excluding 0). Presence also had a mediating effect on aesthetic experience and continued usage intention (β = 0.287, bootstrap 95% confidence interval = 5000 for [0.206, 0.371], excluding 0). Thus, presence plays a partially mediating role between escapist experience, aesthetic experience, and continued usage intention.

**Figure 2:**
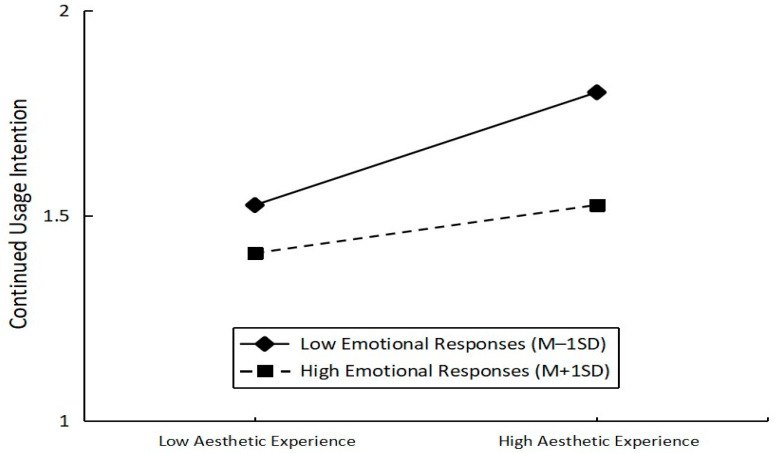
The moderating effect of emotional response on aesthetic experience and continued usage intention.

To test the moderating effect of emotional responses, we used the PROCESS macro model 5. PROCESS macro model 5 is valid for detecting models with moderated mediation. [65]. There was a significant moderating effect of emotional response on the relationship between aesthetic experience and continued usage intention, as shown in Table 4 (β = 0.079, t = 2.158). In order to effectively illustrate the above moderating effect, a slope test was performed. To better explain the moderating effect of emotional responses on aesthetic experience and continued usage intention, we divided emotional responses into two groups: high (M + 1 SD) and low (M − 1 SD) (Figure 2). The results showed that when the affective response was low (mean +1 SD), aesthetic experience had a significant impact on the continued usage intention of VR exhibition users (b = 0.197, t = 3.228, *p* = 0.001). When the emotional response was high (M − 1 SD), the aesthetic experience did not have a significant effect on the continued usage intention of VR exhibition users (b = 0.035, t = 0.568, *p* = 0.570). Thus, we found a significant moderating effect of affective response on aesthetic experience and continued usage intention (β = 0.079, bootstrapping = 5000 with a 95% confidence interval of [0.077, 0.317], excluding 0). Therefore, Hypothesis 4, Hypothesis 5, and Hypothesis 6 are supported.

## 5. Discussion

This study aims to explore the potential mechanisms between escapist experience, aesthetic experience, and users’ continued usage intention for VR exhibitions during COVID-19. Presence is a potential mediator of escapist experience and aesthetic experience, while the affective response is a potential moderator to explain escapist experience on continued usage intention. Users’ escapist and aesthetic experience positively predicted the continued usage intention of VR exhibitions. When users perceive the escapist and aesthetic experiences, they will actively continue to use the VR exhibition. This is consistent with the results of previous studies [9,31]. During the COVID-19 pandemic, there were impacts on human physical and mental health, and when people perceive risks they accordingly assess them and take steps to implement protective behaviors. VR exhibitions enable people to change the way they view exhibitions, break through the limitations of time and space, avoid the risks of offline visits, and allow users to freely enjoy artworks as the ideal state for safe visiting behavior. At the same time, it enhances the exchange, communication, and connection in social activities and makes up for the lack of social interaction in real art activities. When users enter the virtual space, they experience the feeling of being separated from reality, freely enjoying and interacting in the space, appreciating different types of art and different styles of artwork, and feeling the strong aesthetic atmosphere, prompting the intention of wanting to experience it again. In addition, the development and utilization of multiple experience modes and features in the information system provide the quality basis for effective user use.

On the other hand, presence mediates the user’s escapist experience, aesthetic experience, and continued usage intention [31]. Escapist and aesthetic experiences influence users’ continued usage intention through presence. When users perceive an escapist experience, the increase in presence makes them more likely to have continued usage intention. This can be seen as the user being attracted by the rich artwork in the process of art experience that generates a sense of immersion, gradually satisfying the user’s desire for appreciation and participation. As the system continues to improve its functionality, the space for direct user communication is expanded, online participation becomes active, and the active sharing of exhibitions while using VR exhibitions expands participation and experience in virtual art spaces. In particular, features such as co-viewing and commenting in the module increase opportunities for users to interact with the outside world, helping to alleviate the negative emotions generated by COVID-19 and promoting social connection.

Users generate a variety of positive emotions and perceptions during their experience of virtual spaces. High-emotional responses are more likely to make users want to continue the experience. That is, better and higher degrees of emotions and perceptions motivate users to keep using. This is consistent with the results of previous studies [41,66,67]. In other words, when users perceive the aesthetic experience, the VR exhibition provides visual art elements that lead to enjoyment and surprise, which is inseparable from the emotional response of users. In particular, the VR exhibition platform enhances the positive emotional response of users through the personalized design of the display space and the vividness of the exhibited works, that is, the pleasurable emotions after enjoying the VR exhibition to meet their artistic needs. At the same time, it enriches daily life and gives users a sense of subjective well-being, generating a sense of physical and mental benefit, thus improving their health literacy and developing their ability to self-manage their health accordingly. At the social level, the exhibition platform provides the user community with high-quality and scientific information on culture and art and carries out art appreciation activities related to mental health, thus increasing the possibility of continued usage by users. Research has shown that affective responses moderate the relationship between aesthetic experience and continued usage intention. In addition, compared to previous studies, the present study found that emotional responses did not play a moderating role between escapist experience and continued usage intention. This is probably because, in the actual use of VR exhibitions, diverse factors such as the environment, equipment, and users’ operational errors reduce the sense of experience and immersion and do not bring good emotional responses. In particular, due to overuse or wrong use of VR exhibitions, it is easy for users to produce symptoms such as visual fatigue and image vertigo, thus failing to obtain a good escapist experience and emotional responses. Thus, emotional responses did not cause users of the VR exhibit to have continued usage intention when engaging in escapist experiences. In a study of foreign users experiencing VR exhibitions in British museums, Jung et al. found that the escapist experience had a positive impact on continued usage intention and proposed solutions to improve the experience [18]. In other words, it has similar characteristics compared to Chinese users, but this study takes into account the factor of emotional response, and presents more diverse user experience results.

### Limitations and Future Research

There are limitations in this study that provide directions for future research on VR exhibitions. First, the source of the sample data is the Chinese region, and the sample coverage has some limitations. However, the composition of the sample in this study was reasonable, with approximately equal proportions of males and females and a relatively even age distribution, with a certain degree of generalizability and standardization. This investigation is limiting if the scope of the study is expanded. Further studies in the future could also be extended to other countries to investigate this phenomenon. In addition, the medium sample size of this study is one of the limitations. The sample size could be further expanded in future studies. Third, the questionnaires in this study were filled in by the users themselves, and there may be bias in the users’ recall. Fourth, the data sources were not diverse because the sample data were based on questionnaires. Future studies can further explore the relationship between variables through interviews and experimental methods. The present study only examined the moderating role of affective responses, but it can also be used as an antecedent variable to influence continued usage intention. Therefore, this issue can be extended in depth in future studies. In addition, the negative effects of emotional responses can be considered to further investigate the resulting behaviors and performance.

## 6. Conclusions

This study reveals the psychological basis of VR exhibition user experience and explores the factors that influence users’ continued usage intentions. If we want to improve and refine VR exhibitions, we need to understand the psychology of users. This study constructed a moderated mediator model of the relationship between escapist experience, aesthetic experience, presence, emotional response, and continued usage intention. The moderating role of emotional responses was confirmed. From a perceptual point of view, it has significance for the virtual contextualization services of VR exhibitions. From a psychological point of view, it helps users to correctly manage themselves at the emotional level, achieve the desired state of balance, and promote mental health. On the other hand, this study combines experience economy theory and VR exhibition, which expands the boundaries of experience economy theory and the space of application to a certain extent, and helps solve the problem of continued usage intention from the perspective of psychology. At the same time, the mechanism of the influence of escapist and aesthetic experience on users’ continued usage intention was verified by empirical methods. This study enriches the research on VR exhibition themes and provides a new perspective for exploring users’ continued usage intentions. In addition, this study has practical implications. First of all, the aesthetic experience is an important factor affecting the continued usage intention of VR exhibitions. Therefore, in the actual VR exhibition design, emphasis should be placed on artistry and appreciation. Diverse aesthetic elements and stylistic forms can be used in virtual experience programs to visually and psychologically engage users and add artistic themes to the settings. Users can feel the strong art atmosphere in the process of virtual experience so that they can get good emotional responses and promote the sharing and spreading of VR exhibitions. In addition, the visual depth of the screen should be designed at an appropriate level to reduce the user’s visual fatigue and vertigo. Using a larger, clearer screen or device can improve immersion, gain an escapist experience, and help users actively engage in space. Therefore, this study also has an artistic dimension for guidance.

## Figures and Tables

**Table 1:** Descriptive characteristics of the sample (N = 543).

Characteristics		Frequency	The Percentage
Gender	Male	258	47.51
	Female	285	52.49
Age	18 years old and below	119	21.92
	18–26 years old	293	53.96
	27–33 years old	92	16.94
	34–40 years old	26	4.79
	40 years old and above	13	2.39
Education	Highschool graduation	28	5.16
	Junior college degree	173	31.86
	Bachelor degree	201	37.01
	Master degree or above	141	25.97
Frequency of use	Every day	31	5.71
	Once every 1 to 3 days	88	16.20
	Every 4 to 7 days	281	51.75
	More than 7 days	143	26.34

**Table 2:** Pearson Correlation, Descriptive Analysis, Reliability Test.

Variables	M	SD	Cronbach’s α	EE	AE	PS	CUI	ER
EE	3.2252	0.68560	0.873	1				
AE	3.2533	0.81493	0.870	0.489 **	1			
PS	3.2584	0.74852	0.880	0.633 **	0.548 **	1		
CUI	3.2808	0.91980	0.921	0.480 **	0.392 **	0.593 *	1	
ER	3.2205	1.02968	0.906	0.481 **	0.374 **	0.543 **	0.406 **	1

* *p* < 0.05; ** *p* < 0.01. N = 543. M, Mean; SD, Standard Deviation; EE, Escapist Experience; AE, Aesthetic Experience; PS, Presence; CUI, Continued Usage Intention; ER, Emotional Response.

**Table 3:** Direct and indirect impact analysis.

Relationship	β	SE	95% CI		*p*	Results
			Lower	Upper		
	Direct effect					
EE→CUI	0.481	0.051	0.546	0.745	0.000	Supported
AE→CUI	0.374	0.045	0.334	0.511	0.000	Supported
	Indirect effect					
EE→PS→CUI	0.335	0.054	0.235	0.442	0.000	Supported
AE→PS→CUI	0.287	0.043	0.206	0.371	0.000	Supported

N = 543, bootstrapping randomly sampled 5000 times.

**Table 4:** Moderation analysis.

Relationship	β	SE	95% CI		t	*p*	Results
			Lower	Upper			
EE × ER→CUI	–0.024	0.045	–0.112	0.065	–0.525	0.600	Not supported
AE × ER→CUI	0.079	0.037	0.007	0.150	2.158	0.031 *	Supported

* *p* < 0.05. EE, Escapist Experience; AE, Aesthetic Experience; CUI, Continued Usage Intention; ER, Emotional Response.

## Data Availability

The data presented in this study are available on request from the corresponding author. The data are not publicly available due to the privacy restrictions.

## Appendix A

**Table A1:** Questionnaire.

Construct	Items	Description	Source
Escapist experience	EE1	Do you have a feeling of being in another place while experiencing the VR exhibition?	[59,60,61]
	EE2	In the process of experiencing the VR exhibition, there is a feeling of being out of touch with reality.	
	EE3	Forgetting time while experiencing VR exhibition.	
	EE4	In the process of experiencing the VR exhibition, there is a sense of being at different times.	
	EE5	During the visit to the VR exhibition, you can temporarily forget about your daily life.	
	EE6	During the visit to the VR exhibition, it was like stepping back in time.	
	EE7	During the visit to the exhibition, the mood shifted.	
	EE8	It feels like experiencing a new virtual world.	
	EE9	In the process of virtual space experience, it feels like becoming another person.	
	EE10	During the tour of the exhibition, I did not know that time was passing.	
	EE11	During the VR exhibition experience, it felt different from your usual self.	
	EE12	The experience of experiencing the VR exhibition gave me a break from my daily life and rest.	
	EE13	I want to spend more time on VR exhibitions.	
	EE14	Experiencing the VR exhibition immersed me in another art space for a moment.	
	EE15	My experience in virtual space gave me a chance to see myself in a new way.	
Aesthetic experience	AE1	I am very satisfied with the design style of the VR exhibition experience showroom.	[60,62]
	AE2	My visit to the VR exhibition gave me a wonderful and sensual experience.	
	AE3	The lighting in the VR exhibition experience exhibition hall is just right.	
	AE4	The sound effects during the VR exhibition experience are just right.	
	AE5	The video and music of the VR exhibition are well connected.	
	AE6	Experience the VR exhibition, the real and the image perfect fusion.	
	AE7	The environment of the exhibition space is very charming.	
	AE8	The exhibition space is designed with an aesthetic sense.	
	AE9	The atmosphere of the experience space is harmonious.	
Presence	PS1	I felt that the exhibits in these virtual spaces were like real exhibits in front of my eyes.	[2]
	PS2	I feel like I’m looking at artworks in the exhibition hall.	
	PS3	I feel that I also participated in the exhibition.	
	PS4	I feel that the VR exhibition screen exists in real life.	
	PS5	VR exhibition works can bring a multi-sensory immersive experience.	
	PS6	I feel that the exhibits are like being exhibited in front of my eyes.	
	PS7	It feels like moving in the background of a VR exhibition.	
Emotional responses	ER1	I felt pleasure and satisfaction when I had an art experience in the environment of a VR exhibition.	[63]
	ER2	Visit the VR exhibition and feel very leisurely.	
	ER3	I love watching VR exhibitions.	
	ER4	Visiting the VR exhibition was both interesting and enjoyable.	
Continued usage intention	CUI1	I am currently using the VR exhibit and will continue to use it.	[64]
	CUI2	I hope to use VR exhibitions to visit often in the future.	
	CUI3	I would recommend the VR exhibition to those around me.	
	CUI4	I will always share wonderful VR exhibitions so that people around me can also see them.	
